# Functional and Anatomical Connectivity Abnormalities in Cognitive Division of Anterior Cingulate Cortex in Schizophrenia

**DOI:** 10.1371/journal.pone.0045659

**Published:** 2012-09-25

**Authors:** Hao Yan, Lin Tian, Jun Yan, Wei Sun, Qi Liu, Yan-Bo Zhang, Xin-Ming Li, Yu-Feng Zang, Dai Zhang

**Affiliations:** 1 Institute of Mental Health, Peking University, Beijing, China; 2 Key Laboratory for Mental Health, Ministry of Health, Beijing, China; 3 Department of Psychiatry, University of Saskatchewan, Saskatoon, Saskatchewan, Canada; 4 Department of Psychiatry, University of Manitoba, Winnipeg, Manitoba, Canada; 5 Center for Cognition and Brain Disorders and The Affiliated Hospital, Hangzhou Normal University, Hangzhou, Zhejiang, China; 6 National Key Laboratory of Cognitive Neuroscience and Learning, Beijing Normal University, Beijing, China; 7 Peking-Tsinghua Center for Life Sciences, Tsinghua University, Beijing, China; Wake Forest School of Medicine, United States of America

## Abstract

**Introduction:**

Current pathophysiological theories of schizophrenia highlight the role of altered brain functional and anatomical connectivity. The cognitive division of anterior cingulate cortex (ACC-cd) is a commonly reported abnormal brain region in schizophrenia for its importance in cognitive control process. The aim of this study was to investigate the functional and anatomical connectivity of ACC-cd and its cognitive and clinical manifestation significance in schizophrenia by using the resting-state functional magnetic resonance imaging (fMRI) and the diffusion tensor imaging (DTI).

**Methods:**

Thirty-three medicated schizophrenics and 30 well-matched health controls were recruited. Region-of-interest (ROI)-based resting-state functional connectivity analysis and Tract-Based Spatial Statistics (TBSS) were performed on 30 patients and 30 controls, and 24 patients and 29 controls, respectively. The Pearson correlation was performed between the imaging measures and the Stroop performance and scores of the Positive and Negative Syndrome Scale (PANSS), respectively.

**Results:**

Patients with schizophrenia showed significantly abnormal in the functional connectivity and its hemispheric asymmetry of the ACC-cd with multiple brain areas, e.g., decreased positive connectivity with the bilateral putamen and caudate, increased negative connectivity with the left posterior cingulated cortex (PCC), increased asymmetry of connectivity strength with the contralateral inferior frontal gyrus (IFG). The FA of the right anterior cingulum was significantly decreased in patients group (*p* = 0.014). The abnormal functional and structural connectivity of ACC-cd were correlated with Stroop performance and the severity of the symptoms in patients.

**Conclusions:**

Our results suggested that the abnormal connectivity of the ACC-cd might play a role in the cognitive impairment and clinical symptoms in schizophrenia.

## Introduction

Cognitive impairment is the core symptom in schizophrenia [1], and is often more damaging to the function outcome of patients with schizophrenia [2]. With relative sparing of many basic cognitive abilities, deficits of cognitive function in schizophrenia involve higher-order thought processes such as executive control functions [3]. The cognitive division of anterior cingulate cortex (ACC-cd), dorsal and caudal portions of ACC [4], is consistently implicated in executive control processes, such as conflict monitoring, attentional control, and error detection [4]–[7]. Convergent evidence has revealed alteration of ACC in cortical and white matter volume [8], [9], cortical gyrus morphology [10] and activity during performing cognitive tasks [11] in patients with schizophrenia.

Accumulating evidence has suggested that schizophrenia may result from pathological connectivity between brain regions, i.e., the dysconnection hypothesis, which could manifest functionally and/or anatomically [12], [13]. The integrality of the anterior cingulum, the fiber tract of the ACC, has been found disturbed in schizophrenia [14], [15]. In addition, the left-larger-than-right asymmetry of the fractional anisotropy (FA) in cingulum has been found disappeared in schizophrenia [15], [16]. FA is a widely used measure of fiber integrity based on diffusion tensor imaging (DTI), and it is thought to reflect fiber density, axonal diameter, and myelination in white matter [17]. Although the structural connectivity of the ACC in schizophrenia have been widely investigated, the functional connectivity and its hemispheric asymmetry of ACC in patients are not clear yet. With the advent of the functional MRI technology, regions whose blood oxygen level-dependent (BOLD) signal fluctuations show a high degree of temporal correlation are presumed to have functional connectivity [18]. A set of functionally connected regions is referred to as a “functional network” even when participants are not performing any demanding task (i.e., resting state) [19]. The functional networks of ACC in healthy human brain have been mapped out in high detail by using resting-state functional connectivity (RSFC) analysis [20]. Moreover, in a previous study, by using a innovative method we found that the RSFC in the ACC-cd showed significant hemispheric asymmetry in healthy volunteers [21]. Recent studies examining RSFC have demonstrated widespread disrupted inter-region connectivity in patients with schizophrenia [22], [23]. Only one study directly examined the RSFC of ACC in schizophrenia, however, no abnormalities were observed in schizophrenia [24].

ACC is a brain area with highly structural and functional heterogeneity, and ACC-cd is specifically involved in cognitive function. The organization of neural activation and white matter integrity in brain regions may affect the aspects of behavior related to the specific brain regions [25]. Given the impaired executive control function in schizophrenia, the ACC-cd may be prone to be affected in schizophrenia.

To further investigate the functional and structural connectivity of ACC-cd in schizophrenic patients by integrating the functional magnetic resonance imaging (fMRI) and diffusion tensor imaging (DTI). First, we used a method which has been reported elsewhere [21] to investigate the difference in functional connectivity and its hemispheric asymmetries of the ACC-cd between patients with schizophrenia and healthy controls. Second, we used a region of interest (ROI) approach to further test the white matter integrity of ACC-cd in schizophrenia by using the tract-based spatial statistics (TBSS), a DTI post-processing procedure [26]. Third, the Pearson correlation analysis was conducted to characterize the association between the functional and structural connectivity of ACC-cd and executive control impairment and clinical symptoms in schizophrenia.

## Methods

### Ethics Statement

The study was approved by the Medical Research Ethics Committee of the Institute of Mental Health, Peking University. All participants provided written informed consent after complete description of the study.

### Participants

Thirty-three patients with schizophrenia were recruited from the Institute of Mental Health, Peking University. All the patients satisfied the ICD-10 criteria for schizophrenia with paranoid subtype. Exclusion criteria were treatment with electroconvulsive therapy within the last 6 months, a history of seizure disorder or a serious medical illness. All patients were on antipsychotics medication (see Table S1 for details). The medication dosage was converted to chlorpromazine equivalent. The symptom severity of all patients was assessed by a trained and experienced psychiatrist using the Positive and Negative Syndrome Scale (PANSS) within one week of MR scanning. Thirty healthy paid volunteers with no psychotic illness family history were recruited by advertisement. All participants were right-handed, and without a history of head injury, neurological disorder, alcohol or substance abuse. Because of excessive head motion and data missing, 30 patients and 30 controls, 24 patients and 29 controls were remained in the RSFC analysis and TBSS analysis, respectively. There were no significant difference in sex proportion, mean age and years of education between patients and controls (Table 1 and Table S2).

**Table 1:** Demographic, clinical and behavior details.

		Controls (N = 30)		Schizophrenia (N = 30)			
		N	%	N	%	*X* ^2^	*p* (2-tailed)
Sex (female)		12	40.0	13	43.0	0.069	0.793
		Mean	SD	Mean	SD	*t*	*p* (2-tailed)
Age (years)		23.0	3.2	23.1	3.6	0.189	0.851
Education (years)		14.3	2.0	13.7	2.0	−1.245	0.218
Age at onset of illness (years)				19.4	3.2		
Duration of illness (months)				39.0	33.3		
Medication dose (mg)				407.7	240.6		
PANSS_T				67.3	11.8		
PANSS_P				19.4	4.6		
PANSS_N				16.1	4.7		
PANSS_G				31.8	5.4		
**Stroop performance** a							
Accuracy	Word-reading	100%	0	99.9%	0.6%	−1.000	0.322
	Color-naming	99.7%	1.4%	100%	0	1.361	0.179
	Incongruent condition	92.2%	8.5%	87.9%	9.6%	−1.788	0.079
Completion time (seconds)	Word-reading	11.4	2.6	12.5	2.4	1.750	0.086
	Color-naming	15.2	3.3	18.3	4.4	3.027	0.004
	Incongruent condition	31.4	7.6	37.7	9.4	2.825	0.007
	Interference effect	16.2	5.6	19.4	7.4	1.872	0.066

a Sample size in control and schizophrenia groups were 29 and 29, respectively. PANSS_T, total score of PANSS; PANSS_P, score of PANSS positive subscale; PANSS_N, score of PANSS negative subscale; PANSS_G, score of PANSS general psychopathology subscale.

### Executive Control Assessment

Executive control was assessed with the Comalli et al [27]. Stroop version within one week of the MRI scanning. There were three conditions, 30 stimuli in each condition: reading color words (red, blue, yellow and green in Chinese characters) printed in black; naming color of color patches; and naming color of color words printed in a color different from that of the word (i.e., incongruent condition). The measurements for this study were performance accuracy and completion time of each condition. The longer completion time of the incongruent condition compared with that of the color naming of color patches condition was taken as the Stroop interference effect [28].

### Data Acquisition

Imaging was carried out on a Siemens 3.0 Tesla Trio MR scanner at the Third Hospital, Peking University. Head motion was minimized with restraining foam pads. Resting state functional images and DTI images were acquired by using an echo planar imaging (EPI) sequence and a single shot EPI sequence with 20 noncollinear directions, respectively (see parameters in details in Supplementary Materials S1). Participants were instructed to close their eyes, relax, and move as little as possible during the resting-state scan.

### Resting-state Functional Connectivity Analysis

**Figure 1:**
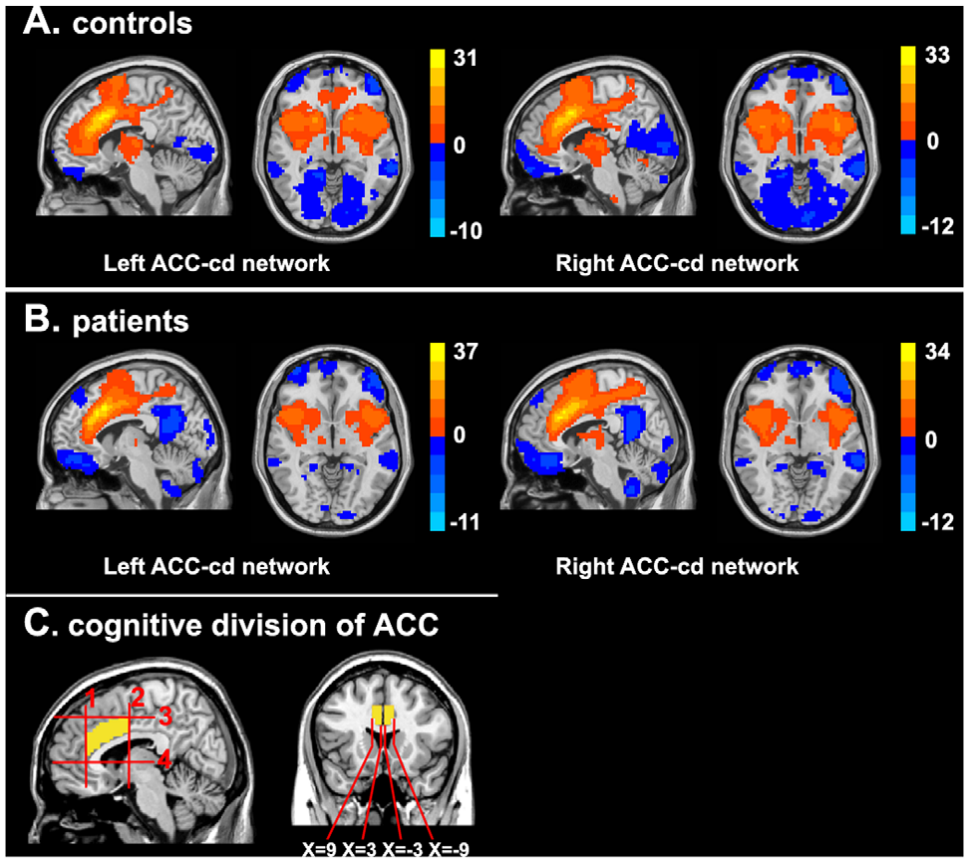
Functional connectivity networks of left and right ACC-cd. (A) Functional connectivity networks of ACC-cd in healthy controls. (B) Functional connectivity networks of ACC-cd in patients with schizophrenia. Significantly positive (warm color) and negative (cool color) connectivity for bilateral ACC-cd seeds are presented in sagittal (X = ±6 for right and left seeds respectively) and axial (Z = 0) view in MNI space, for both LACC-cd and RACC-cd. Color bar indicates the t-value. (C) The regions of interest (ROIs) definition.

As described in our previous study [21], the approach to investigate the functional connectivity and its hemispheric asymmetry of the ACC-cd consisted of five fundamental stages: 1) Creating symmetric brain template and masks for spatial normalization and confounding timeseries extraction; 2) Defining regions of interest (ROIs) of the ACC-cd as two symmetric ACC parcellation regions derived from an anatomical template (see Figure 1C); 3) Preprocessing resting-state fMRI data; 4) Generating individual functional connectivity maps of the left ACC-cd and the right ACC-cd ROIs respectively; 5) Within- and between-group statistical analysis. The fMRI data preprocessing was performed using the software packages of SPM2 (Statistical Parametric Mapping, http://www.fil.ion.ucl.ac.uk/spm/), REST (Resting-State fMRI Data Analysis Toolkit) [29] and AFNI (Analysis of Functional NeuroImages, http://afni.nimh.nih.gov/afni). The stages 1–4 in details can be referred to the Supplementary Materials S1. Excessive movement was found in 3 patients who were then excluded. Data for the remaining 30 patients (17 male) and 30 healthy controls was used in RSFC analysis.

#### Within-group statistical analysis

Within each of patient and control groups, individual RSFC *Z-*maps for left ACC-cd (LACC-cd) and right ACC-cd (RACC-cd) entered into one-sample *t*-test in a voxel-wise manner to determine functional networks of LACC-cd and RACC-cd, respectively. The uncorrected voxel-level *p*<0.001 and cluster size >14 voxels (378 mm^3^) was utilized for both patient and control groups, which corresponded to a corrected *p*<0.05 determined by Monte Carlo simulation (see program AlphaSim by B.D. Ward, http://afni.nimh.nih.gov/pub/dist/doc/manual/AlphaSim.pdf).

Unilateral ACC-cd has functional connectivity with brain regions of both the ipsilateral and contralateral hemispheres. In order to examine the hemispheric asymmetry of functional connectivity of LACC-cd and RACC-cd with brain regions in their ipsilateral and contralateral hemispheres, we generated a new set of *Z*-maps by LR-flipping the individual RACC-cd *Z-*maps, namely LR-flipped RACC-cd *Z-*maps. Then, within each of patient and control groups, the individual LACC-cd and LR-flipped RACC-cd *Z-*maps entered into paired-samples *t*-test in a voxel-wise manner to determine the brain regions that showed significant differences between the LACC-cd and LR-flipped RACC-cd *Z-*maps. The right side of the resultant *t*-map represents the asymmetry of functional connectivity of ACC-cd with its ipsilateral hemisphere (i.e., LACC-cd-left hemisphere [Lcd-LH] vs. RACC-cd-right hemisphere [Rcd-RH]), and the left side represents that with its contralateral hemisphere (i.e., Lcd-RH vs. Rcd-LH). Finally, for each group, the resultant *t*-map was corrected for multiple comparisons to identify the significant hemispheric asymmetry of functional connectivity of ACC-cd, and the regions were thought significant if they met the criteria as follows: (1) significant in RSFC of LACC-cd or LR-flipped RACC-cd in patient and control groups, respectively; (2) corrected *p*<0.05 (uncorrected voxel-level *p*<0.001; cluster size >10 voxels [270 mm^3^]).

#### Between-group statistical analysis

The individual *Z-*maps entered into independent-samples *t*-test in a voxel-wise manner to determine the differences of functional networks of LACC-cd and RACC-cd, respectively, between patient and control groups. The 2 significant within-group RSFC *t*-maps of LACC-cd and RACC-cd were combined as a mask to correct the between-group ACC-cd RSFC map to cluster-level *p*<0.05 determined by Monte Carlo simulation (uncorrected voxel-level *p*<0.001 and cluster size >10 voxels).

To compare the hemispheric asymmetry of RSFC of ACC-cd between groups, we generated a new set of *Z*-maps by subtracting the LR-flipped RACC-cd *Z-*map from the LACC-cd *Z-*map individually, namely LvsflippedRACC-cd *Z-*map. Then the new *Z-*maps entered into independent-samples *t*-test in a voxel-wise manner to determine the hemispheric asymmetry of functional connectivity of ACC-cd between groups. As mentioned above, the right side of the resultant *t-*map presented between-group differences of the asymmetry of RSFC of ACC-cd with its ipsilateral hemisphere, and the left side represented that with the contralateral hemisphere. The corrected hemispheric asymmetry maps of functional connectivity of ACC-cd for patient and control groups were combined as a explicit mask, and the between-group hemispheric asymmetry maps were corrected for multiple comparison within this mask to *p*<0.05 determined by Monte Carlo simulation (uncorrected voxel-level *p*<0.01 and cluster size >13 voxels [351 mm^3^]).

We performed post-hoc ROI analysis to determine the correlation direction of each between-group different brain region in patients and control groups, respectively. The mean *t*-value of each brain region showing between-group difference was extracted from within-group ACC-cd RSFC *t*-maps, and determined the correlation direction by using one-sample *t*-test in each group.

### DTI Imaging Analysis

#### Diffusion tensor calculation

**Figure 2:**
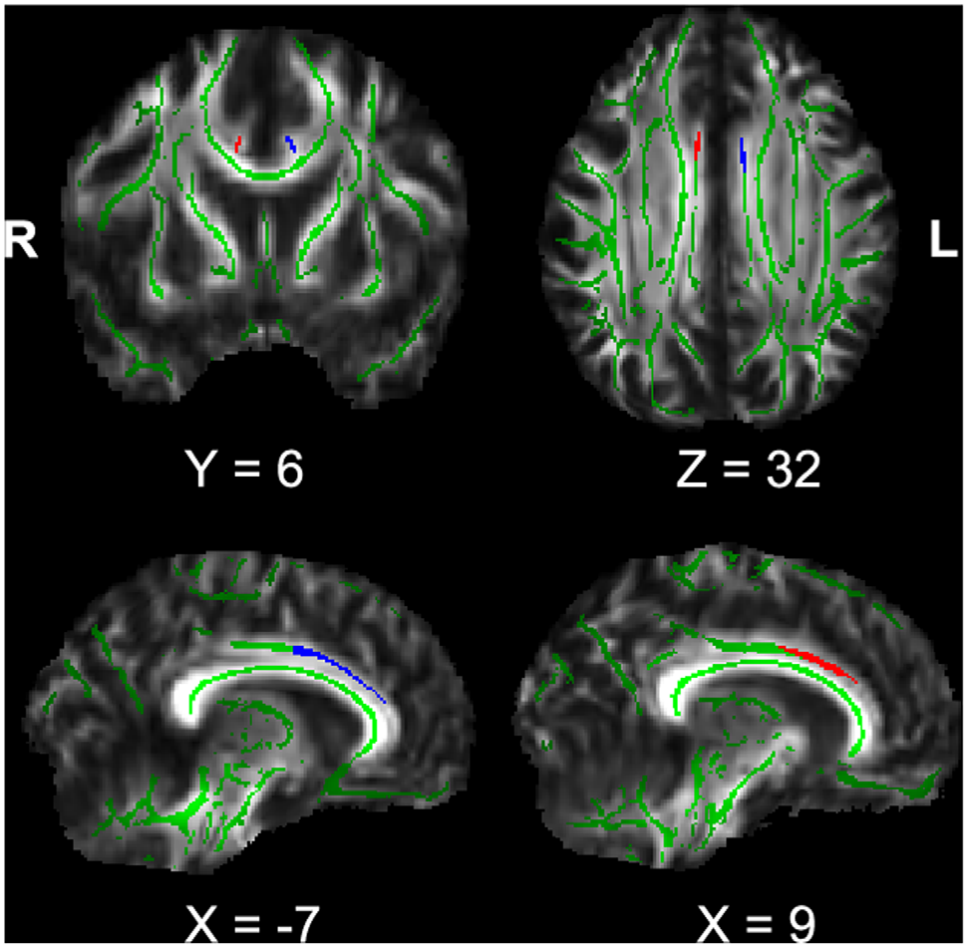
The regions of interest (ROIs) for anterior cingulum. The blue and red regions indicate the left and right anterior cingulum respectively. The green regions indicate the mean FA skeleton for all participants.

Nine patients and one control did not finish the DTI scanning or had missing data because of head motion. Finally, data for 24 patients (16 male) and 29 healthy controls (18 male) entered into DTI data analysis. FSL (FMRIB’s Software Library, http://www.fmrib.ox.ac.uk/fsl/) [30] and TBSS 1.2 [26] were used to perform data preprocessed, fractional anisotropy (FA) calculating and ROI definition for the anterior cingulum (see Figure 2).

Data was preprocessed with FSL (FMRIB’s Software Library, http://www.fmrib.ox.ac.uk/fsl/) [30]. Eddy current correction was applied by using the Eddy Correct program of the FSL package to adjust for the effects of head movement and eddy currents through affine registration. Next, images were brain extracted by using BET program. Fractional anisotropy (FA) maps were calculated by using the DTIfit program and then extracted to prepare for TBSS analysis. TBSS 1.2 [26] was used to do the post-processing of the individual FA maps to get the ROI of the anterior cingulum. All the individual FA maps were aligned into a common space using the nonlinear registration Image Registration Toolkit (IRTK) [31], according to a pre-defined target image (FMRIB58_FA), and then affine-transformed into 1×1×1 mm^3^ MNI152 space. The aligned FA images were then averaged to create a mean FA image. The mean FA image was subsequently thinned to create a mean FA skeleton, which represents the central portion of all the fiber pathways throughout the brain common to both patients and control groups. A threshold FA value of 0.2 was then applied to exclude voxels from the mean FA skeleton that primarily GM or CSF, or with too much cross-subject variability (see the green part in Figure 2). Each individual aligned FA data was then projected onto the mean FA skeleton.

#### TBSS ROI definition

Because our interest was just the fiber tract of the ACC-cd, we did not carry out the voxelwise cross-subject statistics on the skeletonised FA data. ROIs of left and right anterior cingulum (blue and red parts respectively in Figure 2) were defined on the mean FA skeleton following the method adopted in another study [32]. Two WM atlases within FSL (ICBM-DTI-81 parcellation map and JHU WM tractography atlas) were used to guide the placement of the ROIs, and the anterior and posterior borders were the vertical plane at the anterior boundary of the genu of the corpus callosum and the vertical plane through the anterior commissure, respectively. ROI of left anterior cingulum covered 99 voxels and ROI of right cingulum covered 135 voxels.

#### Statistical analysis

Average FA values for each ROI were subsequently calculated for every participant, and then the asymmetry index (AI) of the anterior cingulum was calculated according to the following formula: AI = (right−left)/[0.5(right+left)] [33]. Negative value of AI indicates leftward asymmetry and positive value indicate rightward asymmetry. The individual mean FA values of the left and right cingulum and value of AI entered into independent-samples *t*-test to determine the differences between the patients and controls groups.

### Correlation Analysis between Functional and Structural Connectivity of ACC-cd and Clinical Measures and Stroop Performance

The Pearson correlation between the imaging measures and the cognitive and clinical evaluations were performed. The imaging measures included the RSFC strength of the abnormal ACC-cd network and its hemispheric asymmetry, and FA values of the bilateral anterior cingulum and AI values. For the RSFC measures, we used the peak voxels of the between-group difference of RSFC for the LACC-cd, RACC-cd and hemispheric asymmetry of ACC-cd as the centers and 3 mm as radius to draw spheres. *Z*-value was extracted from each of the spheres by averaging over all voxels within each sphere individually. The cognitive and clinical evaluations included one cognitive measures (i.e., the Stroop interference effect) and 6 clinical measures (i.e., scores of PANSS positive, negative and general psychopathology subscales, onset age, illness duration and medication does.

## Results

### Executive Control Assessment

One patient and one healthy control did not finish the Stroop task. In all three conditions, performance accuracy was not significantly different between patient and control groups. In color naming and incongruent condition, completion time was significantly increased in patient group. The interference effect was increased in patients with schizophrenia with a marginal significance level (*p* = 0.066). (Table 1 and Table S2).

### Functional Connectivity of ACC-cd

#### Connectivity in healthy controls

The patterns of RSFC for the left and right ACC-cd in healthy controls were generally similar. The positive networks of bilateral ACC-cd included the dorsal ACC, dorsal posterior cingulate cortex (dPCC), dorsolateral prefrontal cortex (DLPFC), dorsomedial prefrontal cortex (dmPFC), supplementary motor area (SMA), etc; the negative networks included the orbitofrontal cortex/ventral medial prefrontal cortex (vmPFC), PCC/precuneus (PCC/PCu), amygdala, hippocampal and parahippocampal gyrus, etc (see Figure 1A, Tables S3 and S4).

**Figure 3:**
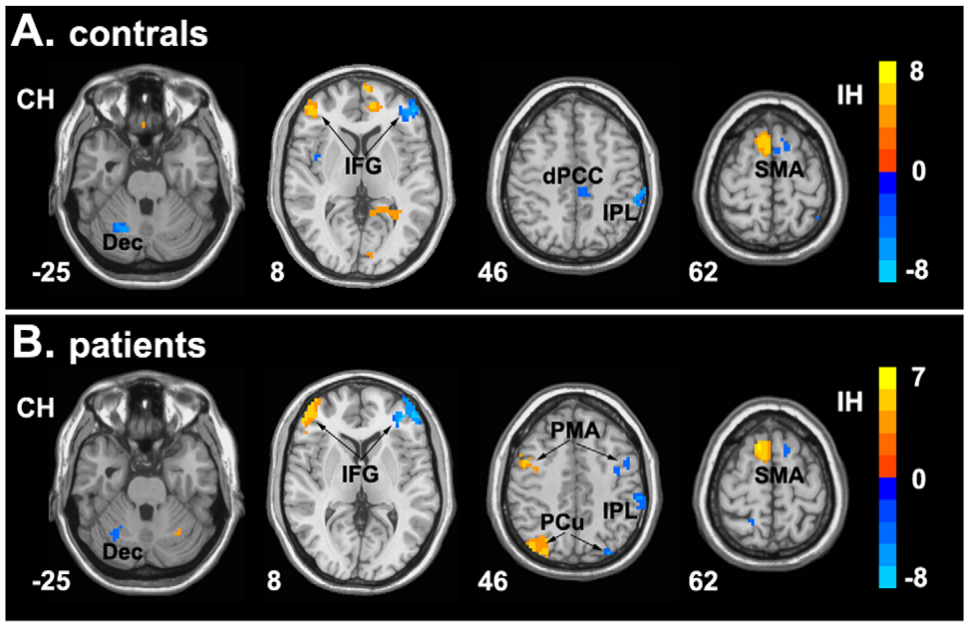
Brain regions showing significant hemispheric asymmetry of functional connectivity with ACC-cd. Hemispheric asymmetry of functional network of ACC-cd in healthy controls (A) and patients with schizophrenia (B). The foci in the right side show significant asymmetric functional connectivity with their ipsilateral ACC-cd, and foci in the left side show significant asymmetric functional connectivity with their contralateral ACC-cd. Color bar indicates the t-value. IH, ipsilateral hemisphere; CH, contralateral hemisphere; Dec, declive; IFG, inferior frontal gyrus; dPCC, dorsal posterior cingulate cortex; IPL, inferior parietal lobe; SMA, supplementary motor ares; PMA, premotor area; PCu, precuneus.

The ACC-cd showed significant right-greater-than-left hemispheric asymmetry of RSFC, regardless of whether the connection was positive or negative. (see Figure 3A and Table S5). Specifically, the right ACC-cd showed significantly greater strength with its ipsilateral inferior parietal lobule (IPL), medial frontal gyrus (MFG), middle frontal gyrus (Mid-FG), etc (see Figure S1A).

#### Altered connectivity in patients

**Figure 4:**
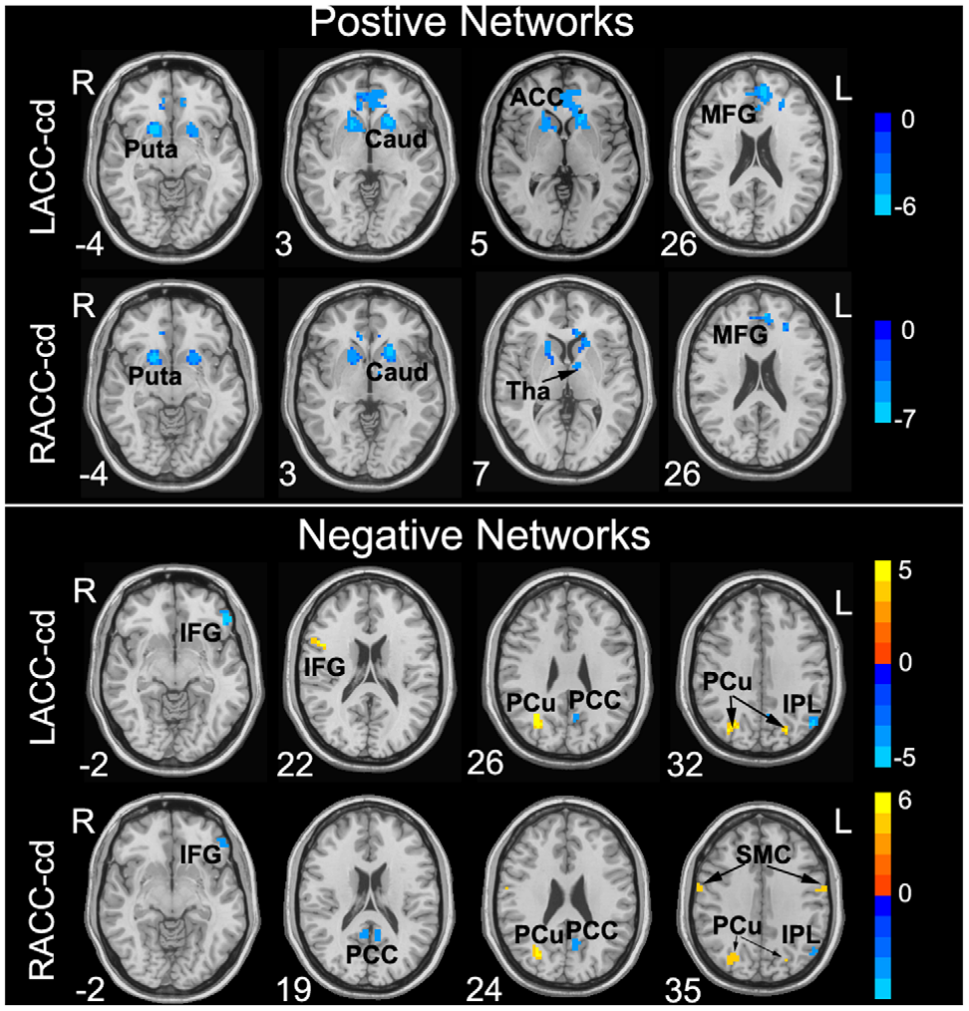
Brain regions showing significantly altered connectivity in positive functional networks and negative functional networks with LACC-cd and RACC-cd in patients with schizophrenia. In the positive networks, cool color indicates decreased or lost positive connectivity in patients. In the negative networks, cool color indicates increased negative connectivity, and warm color indicates decreased or lost negative connectivity. Color bar indicates the t-value. Puta, putamen; Caud, caudate; ACC, anterior cingulate cortex; MFG, medial prefrontal gyrus; Tha, thalamus; IFG, inferior frontal gyrus; PCu, precuneus; PCC, posterior cingulate cortex; IPL, inferior parietal lobule; SMC, sensorimotor cortex.

For patients, the general patterns of RSFC for bilateral ACC-cd (see Figure 1B, Tables S6 and S7) and its hemispheric asymmetry (see Figures 3B and S1B, and Table S8) were similar to those in controls. However, some brain areas showed significant between-group differences. Specifically, in the positive networks, patients showed significantly decreased positive connectivity with bilateral putamen, caudate, left thalamus, dorsal ACC and medial prefrontal cortex (mPFC), and lost positive connectivity (i.e., the connectivity in control group was positive, but in patient group was negative) for ventral ACC and mPFC. In the negative networks, patients showed increased negative connectivity for the left inferior frontal gyrus (IFG), PCC, IPL, decreased negative connectivity for bilateral PCu and lost negative connectivity (i.e., the connectivity in control group was negative, but in patient group was positive) for bilateral sensorimotor cortex (Figure 4, Tables 2 and 3).

**Table 2:** Brain regions showing significant differences for functional connectivity of LACC-cd in patients with schizophrenia compared with healthy controls.

Regions	BA	Coordinates a			t-value	Cluster size b
		*x*	*y*	*z*		
**I. Decreased positive connectivity with the LACC-cd in schizophrenia**						
Left caudate head		−17	19	3	−5.5922	404
Right lentiform nucleus		17	10	−4	−5.8357	114
Left middle frontal gyrus	46	−25	34	26	−4.241	17
**II. Increased negative connectivity with the LACC-cd in schizophrenia**						
Left inferior frontal gyrus	45/47	−51	35	−2	−4.5928	41
Left inferior parietal lobule (angular gyrus)	39	−48	−67	32	−4.0948	40
Left posterior cingulate cortex	23/31	−8	−60	24	−3.6569	12
**III. Decreased negative connectivity with the LACC-cd in schizophrenia**						
Right precuneus	19	29	−66	26	4.917	70
Left precuneus	7	−20	−76	37	4.8283	22
**IV. Lost negative connectivity with the LACC-cd in schizophrenia**						
Right inferior frontal gyrus	44	43	6	22	4.6852	19

BA, Brodmann area;

a The peak voxel in MNI coordinates.

b Minimum cluster size: 10 voxels (270 mm^3^).

**Table 3:** Brain regions showing significant differences for functional connectivity of RACC-cd in patients with schizophrenia compared with healthy controls.

Regions	BA		Coordinates a			t-value	Cluster size b
			*x*	*y*	*z*		
**I. Decreased positive connectivity with the RACC-cd in schizophrenia**							
Left caudate head			−17	19	3	−6.2892	128
Right lentiform nucleus			17	10	−4	−5.9701	114
Left thalamus			−8	−5	7	−4.6637	21
Left medial frontal gyrus		9/32	−25	34	26	−4.4763	19
Left anterior cingulate cortex		24	−8	29	6	−4.4944	16
**II. Increased negative connectivity with the RACC-cd in schizophrenia**							
Left posterior cingulate cortex		23/31	−8	−60	24	−3.9499	38
Left inferior parietal lobule (angular gyrus)		39	−42	−76	40	−4.1309	36
Right posterior cingulate cortex		23/30	9	−51	19	−4.1846	22
Left inferior frontal gyrus		45/47	−51	35	−2	−3.939	19
**III. Decreased negative connectivity with the RACC-cd in schizophrenia**							
Right precuneus		19	29	−69	23	5.4356	81
Left precuneus		7/19	−20	−76	37	4.4353	14
**IV. Lost positive connectivity with the RACC-cd in schizophrenia**							
Left medial frontal gyrus		9/32	−6	46	29	−5.2053	35
Right anterior cingulate cortex		32	9	32	−6	−4.1188	19
**V. Lost negative connectivity with the RACC-cd in schizophrenia**							
Right sensorimotor cortex		3/4	43	−18	56	5.5904	83
Left sensorimotor cortex		3/4	−59	−9	35	4.0602	23
Right sensorimotor cortex		3/4	60	−6	35	3.9375	15

BA, Brodmann area;

a The peak voxel in MNI coordinates.

b Minimum cluster size: 10 voxels (270 mm^3^).

**Figure 5:**
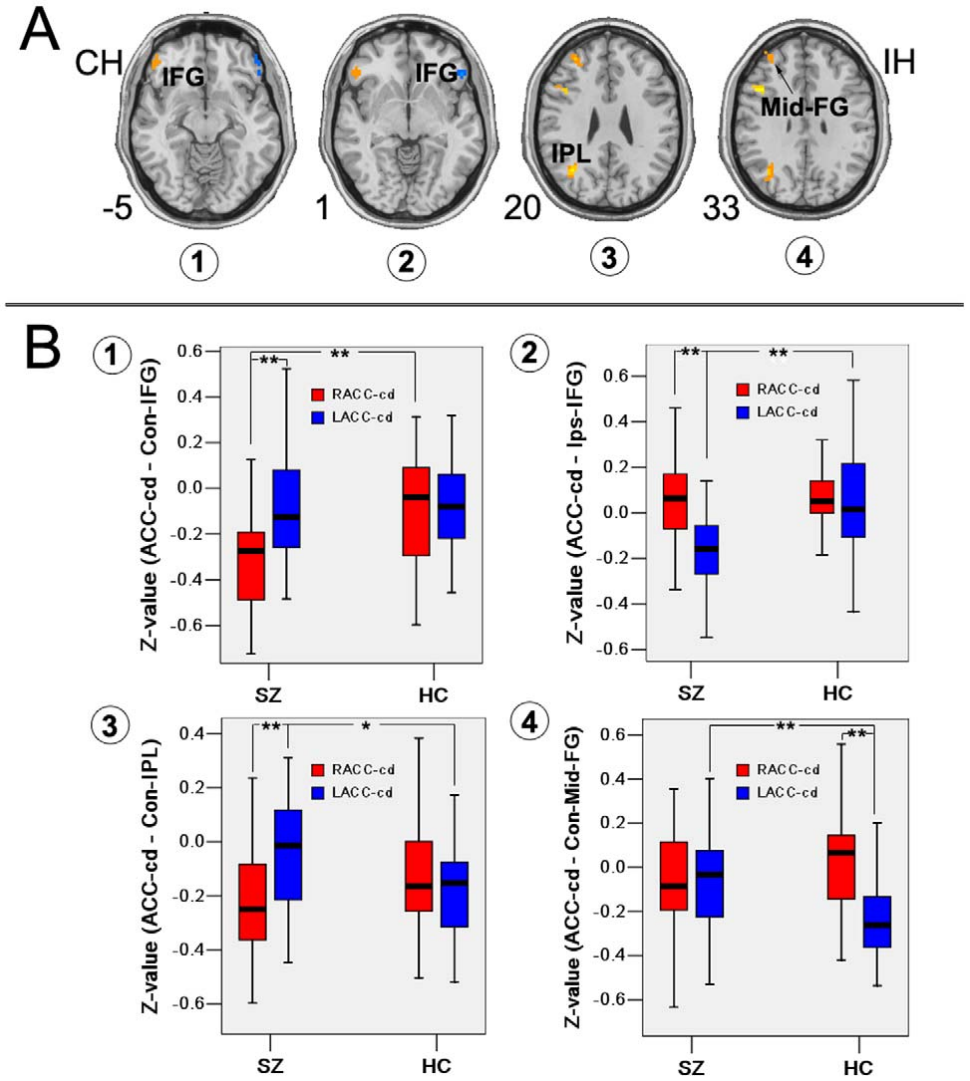
Hemispheric asymmetry of functional network abnormalities in patients with schizophrenia. (A) Brain regions showing significantly altered hemispheric asymmetry of functional connectivity of ACC-cd in patients with schizophrenia. (B) *Z*-values of peak voxel for the contralateral and ipsilateral IFG in the left and right ACC-cd networks in both groups. (1) Increased asymmetry in contralateral IFG. (2) Increased asymmetry in ipsilateral IFG. (3) Increased asymmetry in contralateral IPL. (4) Lost asymmetry in Mid-FG. IFG, inferior frontal gyrus; IPL, inferior parietal lobule; Mid-FG, middle frontal gyrus. *p<0.05; **p<0.001.

In addition, patients showed significant altered hemispheric asymmetry of RSFC of ACC-cd. Patients showed increased asymmetry of connectivity strength with ipsilateral IFG and contralateral IPL, IFG and Mid-FG, decreased asymmetry of connectivity strength with contralateral Mid-FG (see Figure 5 and Table 4).

**Table 4:** Brain regions showing significant differences for hemispheric asymmetry of functional connectivity with ACC-cd in patients with schizophrenia compared with healthy controls.

Regions	BA	Coordinates^a^			t-value	Cluster size^c^
		*x*	*y*	*z*		
**I. Asymmetric connectivity with ipsilateral cerebral hemisphere**						
Inferior frontal gyrus	45/47	48	29	1	−3.4436	24
**II. Asymmetric connectivity with contralateral cerebral hemisphere**						
Inferior parietal lobule	39/40	34	−63	24	4.5441	94
Middle frontal gyrus	46	31	31	20	4.0678	34
Middle frontal gyrus	46	46	34	23	3.374	25
Inferior frontal gyrus	47	49	38	−5	3.2483	24
Middle frontal gyrus	9	43	12	33	5.586	22

BA, Brodmann area.

a The peak voxel in MNI coordinates.

b The positive or negative sign of *x* coordinate has been omitted to avoid confusion, because it does not indicate right or left hemisphere in the table.

c Minimum cluster size: 13 voxels (351 mm^3^).

### Structural Connectivity of ACC-cd

FA values in patients and controls were 0.505±0.047 and 0.525±0.060, respectively, for the left anterior cingulum; 0.433±0.038 and 0.471±0.064, respectively, for the right anterior cingulum. FA value was significantly reduced in patients only in the right anterior cingulum (*p* = 0.014). FA value in the anterior cingulum showed significant leftward asymmetry (*p*<0.001) both in patient (AI = −0.135±0.078) and healthy control (AI = −0.105±0.144) groups. However, there was no significant difference between patients and healthy controls in the left-larger-than-right asymmetry of the FA in anterior cingulum.

### Correlation Analysis between Functional and Structural Connectivity of ACC-cd and Clinical Measures and Stroop Performance

The correlation analysis indicated that the abnormal RSFC strength of the ACC-cd networks and the AI of the FA value in anterior cingulum had relationship with the clinical measures and the Stroop performance in patients (see details in Table 5 and Figure S2 for scatter plots). Taking the left PCC as an example, the larger magnitude of alternation in ACC-cd functional network in patients was associated with the more serious positive symptoms and Stroop performance impairment. The increased hemispheric asymmetries of functional and structural connectivity in ACC-cd were both associated with the more severe negative symptoms in patients with schizophrenia. No significant correlations were found between the connectivity and Stroop performance in healthy controls. Because we tested each of ROIs for correlation analysis between image measures and clinical and cognitive measures, we further corrected the results for multiple-comparison by using Bonferroni correction. However, no results could survive.

**Table 5:** Correlation coefficient between ACC-cd functional networks and clinical measures and Stroop performance in patients with schizophrenia.

Regions^a^	Clinical measures						Stroop
	onset	duration	medication	PANSS_P	PANSS_N	PANSS_G	Interference effect
**Functional connection**							
**(1) LACC-cd network**							
R Puta (P↓)			0.395*				
L PCu (N↓)				0.367*			
**(2) RdACC-cd network**							
L mPFC (P↓)	0.367*						
L Tha (P↓)	0.406*						
L PCC (N↑)				−0.396*			−0.432*
R PCC (N↑)				−0.377*			
L SMC (N↓)	−0.442*	0.410*					
R SMC (N↓)				0.439*			
R SMC (N↓)							−0.410*
**(3) Hemispheric asymmetry network**							
Con-IFG (A↑)					0.415*		.
**Structural connection**							
AI in cingulum					−0.532**		

a Regions with significantly altered functional connectivity with ACC-cd in patients with schizophrenia. P and N in blanket denote the positive and negative networks, respectively; A denotes the asymmetry of functional connectivity of ACC-cd. “↑” denotes the increased connectivity strength in patients; “↓” denotes the decreased connectivity strength in patients. Puta, putamen; PCu, precuneus; mPFC, medial prefrontal cortex; Tha, thalamus; PCC, posterior cingulate cortex; SMC, sensorimotor cortex; Con-IFG, contralateral inferior frontal gyrus; AI, asymmetry index.

* p<0.05,

** p<0.01.

## Discussion

### Overview

In the current study, we investigated the functional and structural dysconnection of ACC-cd in patients with schizophrenia by using resting-state fMRI and DTI data. For the functional connectivity, patients showed significantly disrupted connectivity in both positive and negative networks, as well as its hemispheric asymmetry; for the structural connectivity, patients showed decreased FA in the right anterior cingulum. Moreover, we have demonstrated the behavioral significance of functional and structural dysconnection.

### Disrupted Functional Networks of ACC-cd

For the healthy controls, the RSFC networks of bilateral ACC-cd were consistent with previous study [20], [21]. As a critical brain region involved in cognitive control, the resting-state networks of bilateral cognitive division of ACC comprised two anti-correlated components, i.e. the positive network and negative network, which overlapped well with the two networks often referred to as the task-positive network (TPN) and default mode network (DMN) [34]–[36]. Moreover, although with a relatively small sample size in the current study, the pattern of hemispheric asymmetry of ACC-cd RSFC network well replicated our previous findings in another larger sample of healthy participants [21], especially for the frontal and parietal regions showing right lateralized connection with their ipsilateral ACC-cd. We suggested that the right lateralized subnetwork of ACC-cd intrinsic network supplied new evidence for the right hemisphere dominant fronto-parietal network for cognitive control [37] and spatial orienting [38].

For the patients, the alternations for the functional networks of bilateral ACC-cd were very similar.

#### Positive networks abnormalities

In the positive networks, patients showed reduced positive connectivity in bilateral striatum (i.e., caudate and putamen), left thalamus, bilateral ACC and mPFC; lost positive connectivity in ventral ACC and a more rostral part of mPFC. There is a longstanding hypothesis that schizophrenia is related to dysfunction of corticostriatal circuits [39], [40]. The anatomic study [41] and RSFC study [42] demonstrated multiple functionally segregated circuits linking striatum and different cortex areas, e.g., DLPFC, orbitofrontal cortex, ACC, etc. Our findings of reduced positive connectivity of ACC with striatum together with two previous studies [23], [43], which found reduced positive connectivity of multiple prefrontal areas and DLPFC with striatum, respectively, may support evidence that corticostriatal circuits were disrupted in schizophrenia. Thalamus, as an important component involved in the cortico-basal ganglia-thalamo-cortical loop, connects with ACC through bilateral anterior limb of the internal capsule. The decreased connectivity between ACC and thalamus in the current study may be a result of the structural abnormality of thalamus and the anterior limb of the internal capsule, which has already been reported in previous structural MRI and DTI studies [44], [45].

#### Negative networks abnormalities

The ACC-cd played an important role in cognitive control [4], [6], and bilateral ACC-cd showed anti-correlation, i.e., negative correlation, with key regions involved in DMN, e.g., PCC, PCu, IPL, etc. Patients showed increased negative correlation in PCC and IPL with bilateral ACC-cd. The phenomenon of increased anti-correlation between TPN region and DMN regions was consistent with a previous study [46], in which, they found that increased anti-correlation between the TPN and DMN in schizophrenia. However, another study reported reduced anti-correlation between the TPN region (DLPFC) and DMN regions (mPFC) [47]. The TPN and DMN systems were supposed to support focused external attention and internally directed mentation, respectively [34], [35]. The disturbed (increased or reduced) competition between the TPN and DMN may suggest that the dynamic equilibrium of two brain systems was disrupted in schizophrenia.

However, it was a remarkable fact that, in the ROI-based functional connectivity analysis, the global signal regression method and the interpretation of negative correlation were still an open issue [48], [49], and the findings should be treated with caution.

#### Hemispheric asymmetry of functional network abnormalities

For hemispheric asymmetry of ACC-cd functional network, patients showed similar pattern as controls in the current study and our previous study [21] in some brain areas, e.g., right lateralized connection with ipsilateral IPL, SMA, dPCC and contralateral cerebellum. However, patients showed significant altered hemispheric asymmetry in IFG, Mid-FG and IPL. The neurodevelopment abnormality is one of the etiology hypotheses of schizophrenia [50], the abnormality of lateralization in brain areas including the cortical midline structure may reflect early neurodevelopment defect in schizophrenia [10], [51], [52]. Patients with schizophrenia showed significant abnormalities in brain structural lateralization in multiple brain areas in frontal, parietal, occipital and temporal cortex [16], [53], [54], whereas few study reported the brain lateralization of functional connectivity in schizophrenia. The underlying mechanism of our findings on asymmetry abnormalities of RSFC in patients need to be further investigated.

Recently, only one previous study directly examined the RSFC of ACC in schizophrenia, however, no abnormalities were observed in schizophrenia [24]. The different ROI selection may account for the inconsistent findings. In their study, the ROI of ACC used in RSFC was based on the result of VBM analysis. Therefore, their ROI of ACC was much smaller than ours and only in the right side.

### Disrupted Structural Connectivity of ACC-cd

Compared with healthy controls, patients with schizophrenia showed FA value reduced in the right anterior cingulum. Both controls and patients showed a left-larger-than-right asymmetry for the FA value of anterior cingulum. The findings were consistent with a previous study [55], and partly replicated findings in our previous studies and studies from other groups [14], [15], [56], [57], in which they found the FA was reduced in both side of anterior cingulum. The study of Wang et al. [15] only recruited male patients and the different definitions of ROIs of anterior cingulum in those studies might account for the different findings.

The anterior cingulum is the main fiber tract, with which the ACC contacts with other brain areas, such as DLPFC, premotor area, SMA and parietal cortex [58]. FA is a measure often used in diffusion tensor imaging (DTI) where it is thought to reflect fiber density, axonal diameter, and myelination in white matter [17]. The significant FA reduction in right anterior cingulum may reflect the disruption of the integrity of white matter tracts, which may be the structural foundation of the abnormal functional connectivity of ACC-cd in schizophrenia.

### Possible Effect of Brain Structure on Functional Connectivity

In order to preliminary investigated the possible brain structure on functional connectivity, we additionally extracted the mean gray matter density (GMD) from the ROIs of ACC-cd, and the regions showing significant between group differences of RSFC, and then performed independent-samples t-test between the patients and controls. The Three-dimensional T1-weighted images acquisition and processing could be found elsewhere [59]. We found than the GMD in the LACC-cd was significant reduced in patients (*p* = 0.041), the GMD in ACC-cd showed significant right lateralized but without between-group different (see Table S9). In addition, several abnormal regions of the ACC-cd networks showed significant GMD reduction in the patients group (see Table S10).

We noted a very interesting phenomenon that the anterior cingulum showed a significant greater FA value in the left hemisphere than the right in current and other studies [14]–[16], [60], while the gray matter volume was found greater in the right ACC than in the left one [61], [62], which was verified in our own GMD data. We supposed that larger gray matter in the right ACC-cd might have more fiber projections, which might be one source of the right lateralized functional connectivity strength found in our previous and current studies. In addition, more fiber projections may have a directly influence on the fiber volume and density, which can be reflected by the FA value [63]. As we mentioned in the manuscript, the ROI of left anterior cingulum covered 99 voxels and ROI of right cingulum covered 135 voxels. The more fiber projections/axons might, to some extent, reduce the structural similarity and fiber organization, and then reduce the FA value in the right anterior cingulum.

### Correlation

For the functional connectivity, it was intriguing that all the regions correlated with the symptoms were the abnormal regions in negative networks. Significant correlations between altered functional connectivity and Stroop performance were not observed in control groups, suggesting this relationship was specific to the pathophysiology of the cognitive deficit reflected by the Stroop task in patients. Take the PCC as an example, it showed significantly increased negative connectivity with the ACC-cd and moderately negative correlation with the positive symptom score of PANSS and the interference effect of Stroop test in patients with schizophrenia. Besides the executive control processes, ACC also mediated the mentalizing ability, which is an ability to explain and predict other people’s behaviour by attributing to them independent mental states [64]. In addition, PCC was another key region of neural circuits implementing processes involved in mentalizing [65]. There was empirical evidence that the delusions of alien control and persecution, the thought and language disorganization, and other behavioral symptoms in schizophrenia may be related to the impaired mentalizing ability [66]. Therefore, the disrupted functional connectivity of ACC and PCC may contribute to the positive symptoms in schizophrenia. The TPN and DMN systems were supposed to support focused external attention and internally directed mentation, respectively [34], [35]. The executive control impairment in schizophrenia could be understood in light of attention defect caused by the over competition between the TPN region of ACC and the DMN region of PCC.

Another intriguing finding was that the increased lateralizations of functional and structural connection in ACC-cd were both associated with the more severe negative symptoms in schizophrenic patients. Abnormalities in cerebral lateralization were thought to reflect early neurodevelopmental defects in schizophrenia [51]. Our findings were consistent partly with the few previous studies; the negative symptom in schizophrenia was reported to be associated with the lateralization of brain structure and function [67], [68].

Because no correlation could survive the multiple-comparison correction, our findings on correlation should be interpreted with caution. However, some of the findings with relatively higher level of statistical significance could supply primary evidence for us to propose a more specific hypothesis in the future study.

### Limitation and Future Works

Several limitations to this study are important to consider. First, all the patients received antipsychotic treatment which may have a potential influence on the brain structural and functional connectivity. However, the correlation analysis reveals no significant correlation between the medication and ACC-cd functional and structural connectivity, except for the positive connectivity with the right putamen. Therefore, antipsychotic treatment could not primarily account for the findings. Second, we focused on the cognitive division in ACC. ACC is a heterogeneous region in structure and function. Though the cognitive division is an important and vulnerable sub-division of ACC in schizophrenia, a more comprehensive investigation on its other sub-divisions is necessary in future study. Third, though we tried to infer the effect of the structure on the functional connectivity based on our findings, the current study did not directly establish the relationship between the alternation of functional connectivity and structural connectivity in schizophrenia. Several previous studies supplied us some good examples to combine the structural and functional networks together, by using fMRI, DTI and high-resolution structure MRI data [69], [70]. Future study combining the functional and structural connectivity analysis on patients at different stage of illness will help us better understand whether the dysconnection changes along with the development or treatment of the illness and what’s the causal relationship between the structural and functional connectivity alternations.

In summary, in the current study, we investigated the alternations in the functional networks and white matter integrity of ACC-cd in schizophrenic patients, and then demonstrated that across patients the functional and structural disconnection related to the impaired cognition and symptoms severity. Our findings provided evidence that schizophrenia was a dysconnection syndrome, and the dysconnection of ACC-cd might play a role in the cognitive impairment and symptoms in schizophrenia.

## Supporting Information

Figure S1 **Connectivity strength of LACC-cd and RACC-cd with regions in its ipsilateral cerebral hemisphere.** (A) Connectivity strength in healthy controls. (B) Connectivity strength in patients with schizophrenia. IPL, inferior parietal lobe; IFG, inferior frontal gyrus; MFG, medial frontal gyrus; PCC, dorsal posterior cingulate cortex; SMA, supplementary motor ares; dPCC, dorsal PCC; Mid-FG, middle frontal gyrus; SG, supramarginal gyrus; AG, angular gyrus; PM, premotor area; PCu, precuneus; STG, superior temporal gyrus.
(TIF)

Figure S2 **Representative scatter plots of imaging measures of functional and structural networks against symptoms severity and executive control function in schizophrenic patients with trend lines.** Correlation coefficient (r) was used to indicate the extent of linear relationship (*p*<0.05, uncorrected). L, left; PCC, dorsal posterior cingulate cortex; Con-IFG, contralateral inferior frontal gyrus; AI, asymmetry index; PANSS_P, score of PANSS positive subscale; PANSS_N, score of PANSS negative subscale.
(TIF)

Table S1 **Antipsychotic medications in patients with schizophrenia.**
(DOC)

Table S2 **Demographic, clinical and behavior details for participant in DTI data analysis.**
(DOC)

Table S3 **Brain regions showing significant connectivity with the LACC-cd in healthy controls.**
(DOC)

Table S4 **Brain regions showing significant connectivity with the RACC-cd in healthy controls.**
(DOC)

Table S5 **Brain regions showing significant hemispheric asymmetry of functional connectivity with ACC-cd in healthy controls.**
(DOC)

Table S6 **Brain regions showing significant connectivity with the LACC-cd in patients with schizophrenia.**
(DOC)

Table S7 **Brain regions showing significantly correlation with the RACC-cd in patients with schizophrenia.**
(DOC)

Table S8 **Brain regions showing significant hemispheric asymmetry of functional connectivity with ACC-cd in patients with schizophrenia.**
(DOC)

Table S9 **Grey matter density (GMD) within the ROIs and hemisphere asymmetry of ACC-cd in two hemispheres.**
(DOC)

Table S10 **Significant gray matter density (GMD) reduction in the brain regions showing significant between-group difference in ACC-cd RSFC networks in patients with schizophrenia.**
(DOC)

Supplementary Materials S1 (DOC)
