# Supplementary figures and images for: Functional and Anatomical Connectivity Abnormalities in Cognitive Division of Anterior Cingulate Cortex in Schizophrenia
Source: PLoS One. 2012 Sep 25;7(9):e45659. doi: 10.1371/journal.pone.0045659 (PMC3458074; doi:10.1371/journal.pone.0045659)

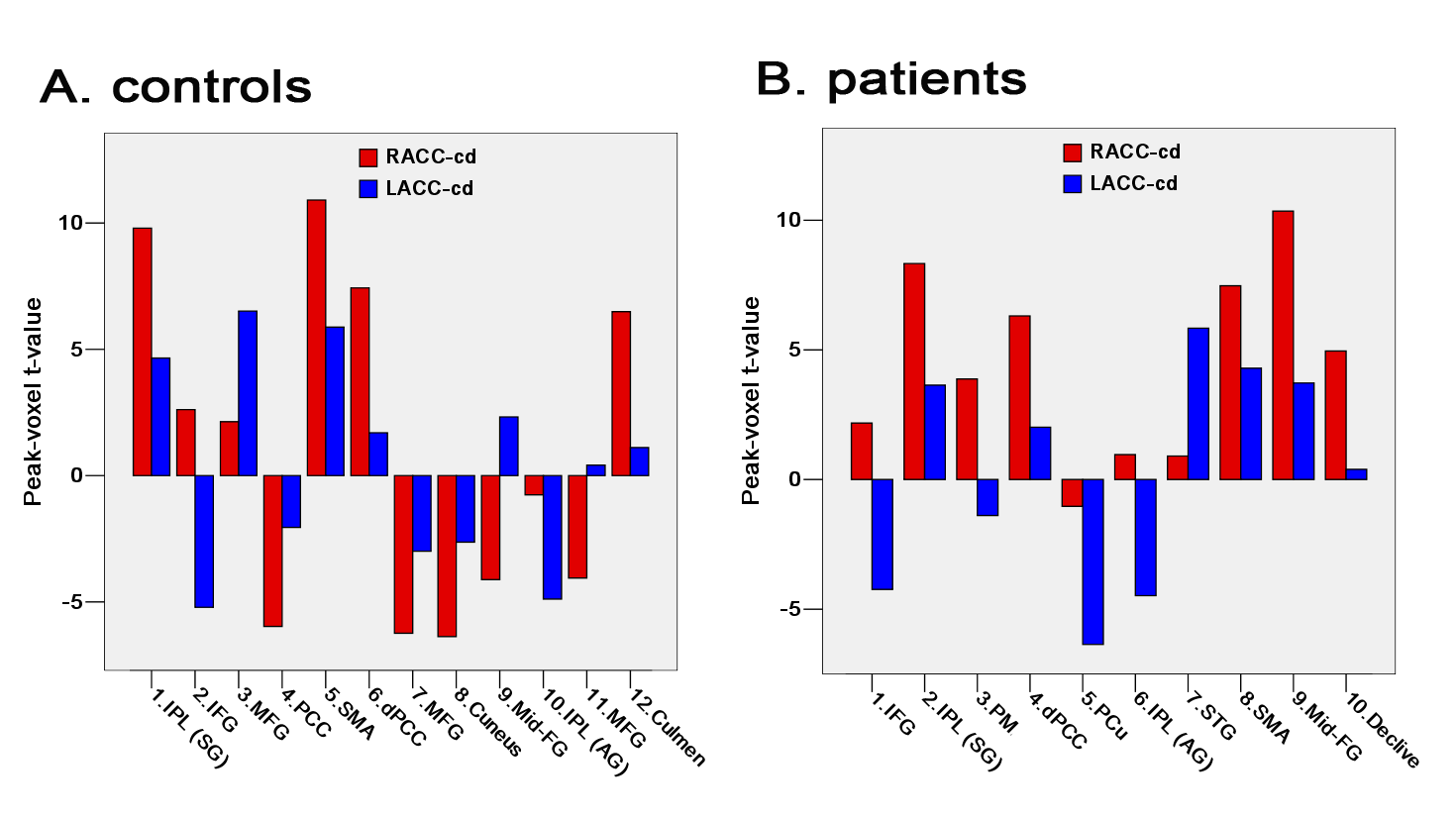

Supplement: Figure S1 — Connectivity strength of LACC-cd and RACC-cd with regions in its ipsilateral cerebral hemisphere. (A) Connectivity strength in healthy controls. (B) Connectivity strength in patients with schizophrenia. IPL, inferior parietal lobe; IFG, inferior frontal gyrus; MFG, medial frontal gyrus; PCC, dorsal posterior cingulate cortex; SMA, supplementary motor ares; dPCC, dorsal PCC; Mid-FG, middle frontal gyrus; SG, supramarginal gyrus; AG, angular gyrus; PM, premotor area; PCu, precuneus; STG, superior temporal gyrus. (TIF) [file pone.0045659.s001.tif]

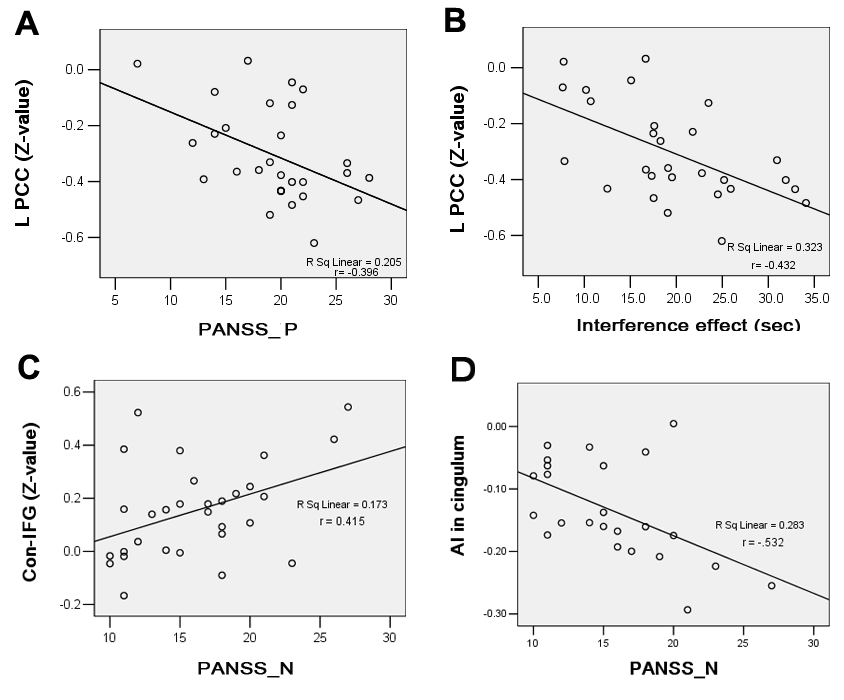

Supplement: Figure S2 — Representative scatter plots of imaging measures of functional and structural networks against symptoms severity and executive control function in schizophrenic patients with trend lines. Correlation coefficient (r) was used to indicate the extent of linear relationship (p<0.05, uncorrected). L, left; PCC, dorsal posterior cingulate cortex; Con-IFG, contralateral inferior frontal gyrus; AI, asymmetry index; PANSS_P, score of PANSS positive subscale; PANSS_N, score of PANSS negative subscale. (TIF) [file pone.0045659.s002.tif]
