# Supplementary material for: Functional and Anatomical Connectivity Abnormalities in Cognitive Division of Anterior Cingulate Cortex in Schizophrenia
Source: PLoS One. 2012 Sep 25;7(9):e45659. doi: 10.1371/journal.pone.0045659 (PMC3458074; doi:10.1371/journal.pone.0045659)
Supplement: Table S1 — Antipsychotic medications in patients with schizophrenia. (DOC) [file pone.0045659.s003.doc]

**Table S1**

Antipsychotic medications in patients with schizophrenia

| Single drug  （N=22） | Combined drug  （N=9） | | |
| --- | --- | --- | --- |
| Atypical APD | Typical APDs | Typical + Atypical APDs | Atypical APDs |
| RIS (14) | PER + THI (1) | HAL + RIS (1) | OLA + CLO (1) |
| OLA (5) |  | HAL + CLO (1) | RIS + OLA (1) |
| QUE (2) |  | HAL + OLA (1) | RIS +QUE (2) |
| PAL (1) |  | PER + QUE + RIS (1) |  |

APD, antipsychotic drug; RIS, risperidone; OLA, olanzapine; QUE, quetiapine; PAL, paliperidone; PER, perphenazine; THI, Thioridazine; HAL, haloperidol; CLO, clozapine;
