# Supplementary material for: Functional and Anatomical Connectivity Abnormalities in Cognitive Division of Anterior Cingulate Cortex in Schizophrenia
Source: PLoS One. 2012 Sep 25;7(9):e45659. doi: 10.1371/journal.pone.0045659 (PMC3458074; doi:10.1371/journal.pone.0045659)
Supplement: Table S2 — Demographic, clinical and behavior details for participant in DTI data analysis. (DOC) [file pone.0045659.s004.doc]

**Table S2**

Demographic, clinical and behavior details for participant in DTI data analysis.

|  | | Controls  (N = 29) | | Schizophrenia  (N = 24) | |  | |
| --- | --- | --- | --- | --- | --- | --- | --- |
|  | | N | % | N | % | *X*^2^ | *p* (2-tailed) |
| Sex (female) | | 11 | 37.0 | 9 | 37.0 | 0.001 | 0.974 |
|  | | Mean | SD | Mean | SD | *t* | *p* (2-tailed) |
| Age (years) | | 22.9 | 3.2 | 22.7 | 3.7 | -0.162 | 0.872 |
| Education (years) | | 14.3 | 2.0 | 14.6 | 2.0 | -1.238 | 0.221 |
| Age at onset of illness (years) | |  |  | 19.0 | 3.2 |  |  |
| Duration of illness (months) | |  |  | 37.8 | 32.1 |  |  |
| Medication dose (mg) | |  |  | 456.8 | 252.8 |  |  |
| PANSS_T | |  |  | 66.7 | 12.2 |  |  |
| PANSS_P | |  |  | 19.0 | 4.4 |  |  |
| PANSS_N | |  |  | 15.6 | 4.5 |  |  |
| PANSS_G | |  |  | 32.0 | 6.0 |  |  |
| **Stroop performance^a^** | |  | |  | |  | |
| Accuracy | Word-reading | 100% | 0 | 99.9% | 0.6% | -1.082 | 0.284 |
|  | Color-naming | 99.6% | 1.4% | 99.6% | 2.0% | -0.125 | 0.901 |
|  | Incongruent condition | 92.0% | 8.6% | 89.2% | 8.9% | -1.171 | 0.247 |
| Completion time (seconds) | Word-reading | 11.3 | 2.6 | 12.7 | 2.5 | 1.856 | 0.069 |
|  | Color-naming | 15.1 | 3.4 | 18.6 | 4.4 | 3.145 | 0.003 |
|  | Incongruent condition | 31.2 | 7.7 | 37.2 | 9.3 | 2.566 | 0.013 |
|  | Interference effect | 16.0 | 5.6 | 18.6 | 7.3 | 1.455 | 0.152 |

^a^ Sample size in control and schizophrenia groups were 28 and 24, respectively. PANSS_T, total score of PANSS; PANSS_P, score of PANSS positive subscale; PANSS_N, score of PANSS negative subscale; PANSS_G, score of PANSS general psychopathology subscale.
