# Supplementary material for: Functional and Anatomical Connectivity Abnormalities in Cognitive Division of Anterior Cingulate Cortex in Schizophrenia
Source: PLoS One. 2012 Sep 25;7(9):e45659. doi: 10.1371/journal.pone.0045659 (PMC3458074; doi:10.1371/journal.pone.0045659)
Supplement: Table S3 — Brain regions showing significant connectivity with the LACC-cd in healthy controls. (DOC) [file pone.0045659.s005.doc]

**Table S3**

Brain regions showing significant connectivity with the LACC-cd in healthy controls

| Regions | BA | Coordinates ^a^ | | | t-value | Cluster  size ^b^ |
| --- | --- | --- | --- | --- | --- | --- |
|  |  | *x* | *y* | *z* |  |  |
| **I. Positive connectivity** | | | | | | |
| Left cingulate gyrus  extending to dPCC, DLPFC, dmPFC, SMA, precuneus, insula, thalamus and basal ganglia | 24/32 | 0 | 24 | 34 | 30.988 | 10826 |
| Right inferior parietal lobule | 40 | 60 | -31 | 31 | 6.4698 | 184 |
| Right culmen |  | 40 | -52 | -36 | 6.4356 | 168 |
| Left declive |  | -20 | -61 | -24 | 5.4408 | 74 |
| Left cerebellar tonsil |  | -34 | -45 | -55 | 4.8282 | 29 |
| **II. Negative connectivity** | | | | | | |
| Right precuneus | 19 | 31 | -76 | 40 | -8.7797 | 4885 |
| Right middle frontal gyrus | 45 | 41 | 31 | 26 | -8.2015 | 450 |
| Right inferior temporal gyrus | 20 | 52 | -9 | -34 | -8.6902 | 439 |
| Right medial frontal gyrus | 11 | 6 | 61 | -17 | -6.0441 | 245 |
| Left middle temporal gyrus | 21 | -62 | -35 | -6 | -8.4919 | 211 |
| Left middle frontal gyrus | 10 | -42 | 54 | 2 | -9.0968 | 185 |
| Right middle frontal gyrus | 47 | 43 | 48 | -8 | -8.7755 | 173 |
| Left fusiform gyrus | 20 | -51 | -3 | -30 | -6.535 | 139 |
| Left middle frontal gyrus | 9 | -45 | 14 | 50 | -4.469 | 109 |
| Right tuber |  | 37 | -72 | -34 | -5.6694 | 98 |
| Right uncus | 36 | 29 | -3 | -40 | -7.1112 | 90 |
| Left superior temporal gyrus | 38 | -34 | 18 | -35 | -5.1646 | 34 |
| Right parahippocampal gyrus | 35 | 17 | -9 | -30 | -5.5307 | 26 |
| Left culmen |  | -37 | -37 | -26 | -4.382 | 24 |
| Left sensorimotor cortex | 3/4 | -54 | -12 | 51 | -4.1828 | 24 |
| Left culmen |  | -3 | -61 | -14 | 4.1067 | 18 |
| Left medial frontal gyrus | 10/11 | -11 | 69 | -3 | -4.2378 | 16 |
| Left fusiform gyrus | 37 | -48 | -55 | -17 | -4.1233 | 15 |
| Left uncus | 20 | -28 | -3 | -43 | -4.3504 | 14 |

BA, Brodmann area; dPCC, dorsal posterior cingulate cortex; DLPFC, dorsolateral prefrontal cortex; dmPFC, dorsal medial prefrontal cortex; SMA, supplementary motor area;

^a^ The peak voxel in MNI coordinates.

^b^ Minimum cluster size: 14 voxels (378 mm^3^).
