# Supplementary material for: Functional and Anatomical Connectivity Abnormalities in Cognitive Division of Anterior Cingulate Cortex in Schizophrenia
Source: PLoS One. 2012 Sep 25;7(9):e45659. doi: 10.1371/journal.pone.0045659 (PMC3458074; doi:10.1371/journal.pone.0045659)
Supplement: Table S4 — Brain regions showing significant connectivity with the RACC-cd in healthy controls. (DOC) [file pone.0045659.s006.doc]

**Table S4**

Brain regions showing significant connectivity with the RACC-cd in healthy controls

| Regions | BA | Coordinates ^a^ | | | t-value | Cluster  size ^b^ |
| --- | --- | --- | --- | --- | --- | --- |
|  |  | *x* | *y* | *z* |  |  |
| **I. Positive connectivity** | | | | | | |
| Right cingulate gyrus  extending to dPCC, DLPFC, dmPFC, SMA, precuneus, insula, thalamus and basal ganglia | 24/32 | 3 | 24 | 34 | 32.603 | 12247 |
| Left culmen |  | -23 | -58 | -27 | 7.0247 | 313 |
| Right cerebellar tonsil |  | 31 | -45 | -52 | 5.4251 | 125 |
| Left declive of vermis |  | 0 | -70 | -18 | 5.3741 | 76 |
| Right premotor cortex | 6 | 43 | -4 | 41 | 5.4638 | 62 |
| **II. Negative connectivity** | | | | | | |
| Right inferior temporal gyrus | 20 | 49 | -6 | -33 | -10.078 | 8270 |
| Left middle temporal gyrus | 21 | -59 | -35 | -3 | -9.7325 | 901 |
| Left medial frontal gyrus | 10/11 | 0 | 48 | -17 | -7.3048 | 777 |
| Left superior frontal gyrus | 9 | -40 | 20 | 53 | -6.4484 | 408 |
| Left middle frontal gyrus | 46 | -42 | 50 | 2 | -11.145 | 237 |
| Right middle temporal gyrus | 21 | 60 | -41 | -7 | -8.4407 | 136 |
| Right middle frontal gyrus | 47 | 43 | 48 | -8 | -8.4258 | 112 |
| Right middle frontal gyrus | 6/8 | 31 | 14 | 47 | -5.6727 | 78 |
| Right sensorimotor cortex | 3/4 | 52 | -12 | 51 | -5.0515 | 67 |
| Left sensorimotor cortex | 3/4 | -59 | -6 | 35 | -5.2298 | 62 |
| Right middle frontal gyrus | 46 | 49 | 37 | 29 | -6.5001 | 48 |
| Right sensorimotor cortex | 3/4 | 60 | -6 | 35 | -4.48 | 28 |
| Right hypothalamus |  | 3 | -1 | -8 | -4.8822 | 17 |
| Left sensorimotor cortex | 3/4 | -48 | -18 | 59 | -4.2043 | 17 |

BA, Brodmann area; dPCC, dorsal posterior cingulate cortex; DLPFC, dorsolateral prefrontal cortex; dmPFC, dorsal medial prefrontal cortex; SMA, supplementary motor area;

^a^ The peak voxel in MNI coordinates.

^b^ Minimum cluster size: 14 voxels (378 mm^3^).
