# Supplementary material for: Functional and Anatomical Connectivity Abnormalities in Cognitive Division of Anterior Cingulate Cortex in Schizophrenia
Source: PLoS One. 2012 Sep 25;7(9):e45659. doi: 10.1371/journal.pone.0045659 (PMC3458074; doi:10.1371/journal.pone.0045659)
Supplement: Table S5 — Brain regions showing significant hemispheric asymmetry of functional connectivity with ACC-cd in healthy controls. (DOC) [file pone.0045659.s007.doc]

**Table S5**

Brain regions showing significant hemispheric asymmetry of functional connectivity with ACC-cd in healthy controls

| Regions | BA | Coordinates ^a^ | | | t-value | Cluster size ^b^ |
| --- | --- | --- | --- | --- | --- | --- |
|  |  | *x* ^b^ | *y* | *z* |  |  |
| **I. Asymmetric connectivity with ipsilateral cerebral hemisphere** | | | | | | |
| 1. Inferior parietal lobule (supramarginal gyrus) | 40 | 56 | -31 | 34 | -6.3437 | 103 |
| 2. Inferior frontal gyrus | 45/46 | 45 | 44 | 10 | -6.3426 | 89 |
| 3. Medial frontal gyrus | 10 | 8 | 50 | 13 | 6.152 | 73 |
| 4. Posterior cingulate cortex | 29/30 | 8 | -51 | 8 | 5.065 | 64 |
| 5. Supplementary motor cortex | 6 | 11 | 4 | 60 | -4.5406 | 54 |
| 6. Dorsal posterior cingulate cortex/ paracentral lobule | 31/4 | 6 | -32 | 50 | -7.1323 | 49 |
| 7. Medial frontal gyrus | 11 | 3 | 48 | -11 | 6.0209 | 22 |
| 8. Cuneus | 17/18 | 6 | -89 | 8 | 5.1763 | 22 |
| 9. Middle frontal gyrus | 9/46 | 45 | 37 | 29 | 5.7591 | 22 |
| 10. Inferior parietal lobule (angular gyrus) | 39 | 42 | -56 | 54 | -4.532 | 17 |
| 11. Medial frontal gyrus | 10 | 6 | 62 | 11 | 5.3904 | 15 |
| **II. Asymmetric connectivity with contralateral cerebellar hemisphere** | | | | | | |
| 12. Culmen |  | 20 | -61 | -27 | -6.1849 | 24 |
| **III. Asymmetric connectivity with contralateral cerebral hemisphere** | | | | | | |
| 1. Middle frontal gyrus | 9/46 | 46 | 37 | 29 | -7.6626 | 166 |
| 2. Supplementary motor cortex | 6 | 6 | 10 | 69 | 7.9036 | 151 |
| 3. Inferior frontal gyrus | 45/46 | 46 | 44 | 10 | 5.8224 | 74 |
| 4. Inferior parietal lobule (supramarginal gyrus) | 40 | 60 | -25 | 17 | -5.2014 | 67 |
| 5. Medial frontal gyrus | 11 | 3 | 39 | -12 | 4.95 | 33 |
| 6. Insula | 13 | 37 | 1 | 5 | -4.7787 | 13 |
| 7. Caudate |  | 9 | 13 | 3 | 4.746 | 10 |

BA, Brodmann area

^a^ The peak voxel in MNI coordinates.

^b^ The positive or negative sign of *x* coordinate has been omitted to avoid confusion, because it does not indicate right or left hemisphere in the table.

^c^ Minimum cluster size: 10 voxels (270 mm^3^).
