# Supplementary material for: Functional and Anatomical Connectivity Abnormalities in Cognitive Division of Anterior Cingulate Cortex in Schizophrenia
Source: PLoS One. 2012 Sep 25;7(9):e45659. doi: 10.1371/journal.pone.0045659 (PMC3458074; doi:10.1371/journal.pone.0045659)
Supplement: Table S6 — Brain regions showing significant connectivity with the LACC-cd in patients with schizophrenia. (DOC) [file pone.0045659.s008.doc]

**Table S6**

Brain regions showing significant connectivity with the LACC-cd in patients with schizophrenia

| Regions | BA | Coordinates ^a^ | | | t-value | Cluster  size ^b^ |
| --- | --- | --- | --- | --- | --- | --- |
|  |  | *x* | *y* | *z* |  |  |
| **I. Positive connectivity** | | | | | | |
| Left cingulate gyrus  extending to dPCC, DLPFC, SMA, precuneus, insula, thalamus and basal ganglia | 24/32 | -6 | 15 | 39 | 36.37 | 10553 |
| Left middle frontal gyrus | 9/46 | -31 | 40 | 29 | 17.969 | 548 |
| Left culmen |  | -34 | -49 | -36 | 8.4319 | 144 |
| Right culmen |  | 34 | -49 | -36 | 6.3844 | 41 |
| Right cerebellar tonsil |  | 31 | -48 | -55 | 5.9053 | 25 |
| Left inferior semilunar lobule |  | -23 | -59 | -62 | 5.0542 | 15 |
| **II. Negative connectivity** | | | | | | |
| Left pyramis |  | -37 | -66 | -43 | -9.9191 | 1699 |
| Right posterior cingulate cortex | 30 | 6 | -51 | 16 | -10.451 | 1341 |
| Left culmen |  | -8 | -48 | -8 | -9.4961 | 1096 |
| Right middle frontal gyrus | 8 | 31 | 20 | 61 | -8.5422 | 991 |
| Left angular gyrus | 39 | -37 | -64 | 35 | -10.772 | 901 |
| Left inferior temporal gyrus | 21 | -62 | -12 | -21 | -8.6798 | 651 |
| Right angular gyrus | 39 | 49 | -63 | 29 | -11.812 | 633 |
| Right fusiform gyrus | 20 | 49 | -6 | -30 | -8.1045 | 568 |
| Left middle frontal gyrus | 46 | -42 | 51 | -5 | -10.368 | 357 |
| Right cerebellar tonsil |  | 3 | -54 | -52 | -6.7859 | 163 |
| Left superior frontal gyrus | 10 | -14 | 65 | 19 | -4.9857 | 44 |
| Right parahippocampal gyrus | 36 | 29 | -32 | -19 | -6.8715 | 32 |
| Right cuneus | 19 | 17 | -87 | 33 | -4.905 | 24 |

BA, Brodmann area; dPCC, dorsal posterior cingulate cortex; DLPFC, dorsolateral prefrontal cortex; SMA, supplementary motor area;

^a^ The peak voxel in MNI coordinates.

^b^ Minimum cluster size: 14 voxels (378 mm^3^).
