# Supplementary material for: Functional and Anatomical Connectivity Abnormalities in Cognitive Division of Anterior Cingulate Cortex in Schizophrenia
Source: PLoS One. 2012 Sep 25;7(9):e45659. doi: 10.1371/journal.pone.0045659 (PMC3458074; doi:10.1371/journal.pone.0045659)
Supplement: Table S7 — Brain regions showing significantly correlation with the RACC-cd in patients with schizophrenia. (DOC) [file pone.0045659.s009.doc]

**Table S7**

Brain regions showing significantly correlation with the RACC-cd in patients with schizophrenia

| Regions | BA | Coordinates ^a^ | | | t-value | Cluster  size ^b^ |
| --- | --- | --- | --- | --- | --- | --- |
|  |  | *x* | *y* | *z* |  |  |
| **I. Positive connectivity** | | | | | | |
| Left cingulate gyrus  extending to dPCC, DLPFC, SMA, precuneus, insula, thalamus and basal ganglia | 24/32 | 0 | 12 | 39 | 33.248 | 10017 |
| Left insula | 13 | -40 | 4 | 5 | 14.137 | 2494 |
| Left middle frontal gyrus | 9/46 | -31 | 40 | 29 | 16.155 | 464 |
| Left culmen |  | -37 | -52 | -33 | 10.539 | 251 |
| Left cerebellar tonsil |  | -34 | -48 | -58 | 5.6097 | 90 |
| Right culmen |  | 34 | -49 | -36 | 5.1771 | 21 |
| Right cerebellar tonsil |  | 31 | -42 | -55 | 5.3157 | 18 |
| **II. Negative connectivity** | | | | | | |
| Left medial frontal gyrus | 10 | -6 | 42 | -11 | -11.365 | 2603 |
| Right uvula |  | 14 | -84 | -35 | -10.151 | 1847 |
| Left angular gyrus | 39 | -37 | -64 | 35 | -13.513 | 1022 |
| Left inferior temporal gyrus | 20 | -54 | -3 | -36 | -9.9242 | 892 |
| Right middle temporal gyrus | 21 | 60 | -4 | -24 | -9.0657 | 633 |
| Right superior temporal gyrus | 39 | 49 | -63 | 26 | -9.0463 | 482 |
| Left middle frontal gyrus | 46/47 | -45 | 48 | -5 | -11.452 | 461 |
| Right cerebellar tonsil |  | 6 | -48 | -52 | -7.4711 | 214 |
| Right middle frontal gyrus | 46/47 | 40 | 51 | -8 | -9.8518 | 208 |
| Right parahippocampal gyrus | 36 | 29 | -32 | -19 | -6.6277 | 43 |

BA, Brodmann area; dPCC, dorsal posterior cingulate cortex; DLPFC, dorsolateral prefrontal cortex; SMA, supplementary motor area;

^a^ The peak voxel in MNI coordinates.

^b^ Minimum cluster size: 14 voxels (378 mm^3^).
