# Supplementary material for: Functional and Anatomical Connectivity Abnormalities in Cognitive Division of Anterior Cingulate Cortex in Schizophrenia
Source: PLoS One. 2012 Sep 25;7(9):e45659. doi: 10.1371/journal.pone.0045659 (PMC3458074; doi:10.1371/journal.pone.0045659)
Supplement: Table S8 — Brain regions showing significant hemispheric asymmetry of functional connectivity with ACC-cd in patients with schizophrenia. (DOC) [file pone.0045659.s010.doc]

**Table S8**

Brain regions showing significant hemispheric asymmetry of functional connectivity with ACC-cd in patients with schizophrenia

| Regions | BA | Coordinates ^a^ | | | t-value | Cluster size ^b^ |
| --- | --- | --- | --- | --- | --- | --- |
|  |  | *x* ^b^ | *y* | *z* |  |  |
| **I. Asymmetric connectivity with ipsilateral cerebral hemisphere** | | | | | | |
| 1. Inferior frontal gyrus | 10/46 | -37 | 41 | 7 | -7.8048 | 270 |
| 2. Inferior parietal lobule (supramarginal gyrus) | 40 | -56 | -32 | 42 | -5.5276 | 99 |
| 3. Premotor area | 6 | -37 | -2 | 55 | -4.7655 | 47 |
| 4. Dorsal posterior cingulate cortex/ precuneus | 31/5 | -6 | -38 | 55 | -4.917 | 44 |
| 5. Precuneus | 19 | -28 | -82 | 42 | -4.7478 | 38 |
| 6. Inferior parietal lobule (angular gyrus) | 39 | -31 | -66 | 29 | -4.9727 | 22 |
| 7. Superior temporal gyrus | 42 | -65 | -34 | 20 | 5.3952 | 21 |
| 8. Supplementary motor cortex | 6 | -11 | 10 | 63 | -4.6103 | 20 |
| 9. Middle frontal gyrus | 10/46 | -28 | 37 | 20 | -5.6798 | 13 |
| **II. Asymmetric connectivity with contralateral cerebellar hemisphere** | | | | | | |
| 10. Declive |  | 29 | -61 | -30 | -5.7507 | 45 |
| **III. Asymmetric connectivity with contralateral cerebral hemisphere** | | | | | | |
| 1. Inferior frontal gyrus | 45/47 | 46 | 32 | 1 | 6.7542 | 251 |
| 2. Inferior parietal lobule (angular gyrus) | 39 | 34 | -63 | 29 | 6.989 | 243 |
| 3. Supplementary motor cortex | 6 | 11 | 10 | 63 | 6.2731 | 148 |
| 4. Premotor area | 6 | 43 | 2 | 49 | 5.4561 | 70 |
| 5. Superior temporal gyrus | 22/42 | 66 | -27 | 17 | -5.9919 | 59 |
| 6. Posterior cingulate cortex | 23 | 6 | -60 | 18 | 4.7076 | 25 |
| 7. Middle frontal gyrus | 44 | 43 | 12 | 33 | 5.6198 | 24 |
| 8. Precuneus | 7 | 17 | -53 | 57 | -4.8663 | 14 |
| **IV. Asymmetric connectivity with ipsilateral cerebellar hemisphere** | | | | | | |
| 9. Culmen |  | -28 | -61 | -33 | 5.5874 | 29 |

BA, Brodmann area

^a^ The peak voxel in MNI coordinates.

^b^ The positive or negative sign of *x* coordinate has been omitted to avoid confusion, because it does not indicate right or left hemisphere in the table.

^c^ Minimum cluster size: 10 voxels (270 mm^3^).
