# Supplementary material for: Functional and Anatomical Connectivity Abnormalities in Cognitive Division of Anterior Cingulate Cortex in Schizophrenia
Source: PLoS One. 2012 Sep 25;7(9):e45659. doi: 10.1371/journal.pone.0045659 (PMC3458074; doi:10.1371/journal.pone.0045659)
Supplement: Table S9 — Grey matter density (GMD) within the ROIs and hemisphere asymmetry of ACC-cd in two hemispheres. (DOC) [file pone.0045659.s011.doc]

**Table S9**

Grey matter density (GMD) within the ROIs and hemisphere asymmetry of ACC-cd in two hemispheres

|  | | Mean (SD) | | 2-sample *t*-test | |
| --- | --- | --- | --- | --- | --- |
|  |  | SZ (n=30) | HC (n=30) | t (df = 58) | p (2-tailed) |
| GMD value | LACC-cd | 0.693 (0.043) | 0.720 (0.054) | -2.094 | **0.041** |
|  | RACC-cd | 0.741 (0.036) | 0.761 (0.047) | -1.766 | 0.083 |
| Asymmetry index (AI) | | 0.138 (0.093) | 0.120 (0.114) | 0.670 | 0.505 |
