# Supplementary material for: Functional and Anatomical Connectivity Abnormalities in Cognitive Division of Anterior Cingulate Cortex in Schizophrenia
Source: PLoS One. 2012 Sep 25;7(9):e45659. doi: 10.1371/journal.pone.0045659 (PMC3458074; doi:10.1371/journal.pone.0045659)
Supplement: Table S10 — Significant gray matter density (GMD) reduction in the brain regions showing significant between-group difference in ACC-cd RSFC networks in patients with schizophrenia. (DOC) [file pone.0045659.s012.doc]

**Table S10**

Significant gray matter density (GMD) reduction in the brain regions showing significant between-group difference in ACC-cd RSFC networks in patients with schizophrenia

| **Regions^a^** | ***t*-value** | | **df** | | **Sig. (2-tailed)** |
| --- | --- | --- | --- | --- | --- |
| **Regions in the LACC-cd RSFC network** | | | | | |
| Left caudate head I | | -2.266 | | 58 | .027 |
| Left inferior frontal gyrus II | | -2.188 | | 58 | .033 |
| Left angular gyrus II | | -2.307 | | 58 | .025 |
| Left precuneus III | | -2.436 | | 58 | .019 |
| **Regions in the RACC-cd RSFC network** | | | | | |
| Left inferior frontal gyrus II | | -2.023 | | 58 | .048 |

^a^ The Roman numerals after the name of brain regions indicated the altered direction of the RSFC in patients, which were the same as those in the Tables 2 and3 in Manuscript.
