# Supplementary material for: Functional and Anatomical Connectivity Abnormalities in Cognitive Division of Anterior Cingulate Cortex in Schizophrenia
Source: PLoS One. 2012 Sep 25;7(9):e45659. doi: 10.1371/journal.pone.0045659 (PMC3458074; doi:10.1371/journal.pone.0045659)
Supplement: Supplementary Materials S1 — (DOC) [file pone.0045659.s013.doc]

**Supplementary Materials**

**Methods**

**Data acquisition**

Resting state functional images were acquired by using an echo planar imaging (EPI) sequence with the following parameters: time repetition [TR]/time echo [TE] = 2000/30 ms, thickness/gap = 4/0.8 mm, matrix = 64 × 64, field of view [FOV] = 220 × 220 mm2, acquisition voxel size = 3.4 × 3.4 × 4.8 mm3, flip angle = 90º, slices = 30, measurements=210. Single shot EPI DTI was acquired in 20 noncollinear directions using the following parameters: TR/TE= 5300/92 ms, thickness/gap = 3/0.3 mm, matrix = 128 × 128, FOV = 230 × 230 mm2, acquisition voxel size = 1.8 × 1.8 × 3.0 mm3, b value=1000 s/mm2, number of excitation = 4, slices = 40, and the scanning time for this sequence was 7 min 43 sec. Other scans included were high-resolution T1-weighted (MP-RAGE) anatomical images and a few of cognitive task scans, which were not used in the current study. Of note, the sequence of the scans was identical in all participants, and the resting-state scan always followed the anatomical images scan which occurred first in the session.

**Resting-state functional connectivity analysis**

The method to investigate the functional connectivity and its hemispheric asymmetry of the ACC-cd has been described elsewhere [1]. Our approach consists of five fundamental stages, and the first 3 steps were the preparations for the functional connectivity analysis.

1) Creating symmetric template and masks

In order to create a symmetric echo planar imaging (EPI) template for spatial normalization, the EPI template available in the SPM2 (Statistical Parametric Mapping) software package (http://www.fil.ion.ucl.ac.uk/spm/) was flipped horizontally in the midsagittal plane (X = 0), i.e., LR-flipped, then an average image from the original and the LR-flipped EPI templates was created. In addition, the symmetric masks of the entire brain, white matter (WM) and cerebrospinal fluid (CSF) were created for the nuisance covariates extraction during the functional connectivity analysis. The processes of creating the masks were as follows. Firstly, the a priori probability maps of the entire brain, WM and CSF provided by the SPM2 package were thresholded to ensure 60%, 90% and 50% tissue type probability, respectively. Then the symmetric binary masks of the entire brain, WM and CSF were created by merging the original binary maps and their LR-flipped counterparts.

2) Definition of regions of interest (ROIs)

The symmetric ROIs of bilateral ACC-cd were defined according to the Automated Anatomical Labeling (AAL) template [2]. The bilateral ROIs were symmetrical and their borders were determined based on the previous literatures (see Fig. 1C). In brief, the anterior border was the vertical plane at the anterior boundary of the genu of the corpus callosum, and the posterior border was the vertical plane through the anterior commissure [3]; the inferior border was the horizontal plane through the anterior boundary of the genu of the corpus callosum [4,5]. And we restricted the ROIs between X coordinates of (-9, -3) and (3, 9), respectively, to keep the ROIs mostly in the gray matter.

3) fMRI data preprocessing

Initial fMRI data preprocessing, including slice timing, realignment, spatial normalization was performed using SPM2 software after the first 10 volumes were discarded. Each participant’s head motion parameters were examined. Datasets with head motion of more than 3 mm maximum translation or 3° maximum rotation in any direction were discarded. Excessive movement was found in 3 patients, who were then excluded. Data for the remaining 30 patients (17 male) and 30 healthy controls was used in the subsequent analysis. During the spatial normalization each dataset was transformed into the symmetric EPI template described above. Linear detrending and temporal bandpass filtering (0.01 - 0.08 Hz) were carried out using a homemade software package named REST (Resting-State fMRI Data Analysis Toolkit) [6]. Spatial smoothing was carried out with a 6-mm full-width half maximum (FWHM) Gaussian filter using AFNI (Analysis of Functional NeuroImages, <http://afni.nimh.nih.gov/afni>).

4) Generating individual functional connectivity maps of the left ACC-cd and the right ACC-cd ROIs

Functional connectivity analyses were carried out by using the partial correlation as implemented in AFNI program 3dfim+ (see program 3dfim+ by B.D. Ward, http://afni.nimh.nih.gov/pub/dist/doc/manual/3dfim+.pdf). Functional connectivity analyses for the bilateral ACC-cd ROIs (see Figure 1C) were carried out by calculating the partial correlation coefficient between the timeseries of each ACC-cd ROI and that of each voxel of the brain, with nine nuisance covariates including six head motion parameters, global signal, white matter (WM), and cerebrospinal fluid (CSF). For each participant, the timeseries for left ACC-cd (LACC-cd) and right ACC-cd (RACC-cd), was obtained by averaging the fMRI timeseries of all voxels within each ROI. The timeseries of nuisance covariates were extracted from the symmetric masks of the entire brain, WM and CSF, respectively, by averaging over all voxels within the masks. To improve normality, these partial correlation coefficients were then transformed to Z-values using the Fisher’s z-transformation.

**Reference**

1. Yan H, Zuo XN, Wang D, Wang J, Zhu C, et al. (2009) Hemispheric asymmetry in cognitive division of anterior cingulate cortex: a resting-state functional connectivity study. Neuroimage 47: 1579-1589.

2. Tzourio-Mazoyer N, Landeau B, Papathanassiou D, Crivello F, Etard O, et al. (2002) Automated anatomical labeling of activations in SPM using a macroscopic anatomical parcellation of the MNI MRI single-subject brain. Neuroimage 15: 273-289.

3. Bush G, Luu P, Posner MI (2000) Cognitive and emotional influences in anterior cingulate cortex. Trends Cogn Sci 4: 215-222.

4. Margulies DS, Kelly AM, Uddin LQ, Biswal BB, Castellanos FX, et al. (2007) Mapping the functional connectivity of anterior cingulate cortex. Neuroimage 37: 579-588.

5. Koski L, Paus T (2000) Functional connectivity of the anterior cingulate cortex within the human frontal lobe: a brain-mapping meta-analysis. Exp Brain Res 133: 55-65.

6. Song XW, Dong ZY, Long XY, Li SF, Zuo XN, et al. (2011) REST: a toolkit for resting-state functional magnetic resonance imaging data processing. PLoS One 6: e25031.
